# Special Issue “Rheumatic Diseases: Pathophysiology, Targeted Therapy, Focus on Vascular and Pulmonary Manifestations 2022”

**DOI:** 10.3390/ph16050652

**Published:** 2023-04-27

**Authors:** Barbara Ruaro, Murray Baron, Edoardo Rosato, Romeo Martini, Marco Confalonieri

**Affiliations:** 1 Pulmonology Unit, Department of Medical Surgical and Health Sciences, University Hospital of Cattinara, University of Trieste, 34149 Trieste, Italy; 2 Division of Rheumatology, Jewish General Hospital, McGill University, Montreal, QC 3755, Canada; 3 Department of Translational and Precision Medicine, Sapienza University of Rome, 00185 Rome, Italy; 4 Unit of Angiology, AULSS 1 Dolomiti, Ospedale San Martino, 32100 Belluno, Italy

This Special Issue, titled “Rheumatic Diseases: Pathophysiology, Targeted Therapy, Focus on Vascular and Pulmonary Manifestations”, aims to demonstrate recent and new advances and future trends in the field of rheumatic diseases. Rheumatic diseases represent a heterogeneous group of serious autoimmune disorders. This Special Issue provides an overview of the diversity and complexity of vascular and pulmonary manifestations of rheumatic diseases and highlights gaps in our knowledge of treatments for these diseases. Indeed, despite their significant morbidity, our understanding of their pathogenesis is limited [1,2,3]. The eight published articles highlight different aspects of rheumatic diseases and the difficulty of managing them. The first evaluated the tolerability and safety of nintedanib (NTD) in patients with idiopathic pulmonary fibrosis (IPF). This study confirmed that NTD can stabilize the value of forced vital capacity, an important lung function test parameter, in patients with IPF, even in a real clinical situation. Moreover, this observation confirms the safety of NTD, even in patients treated with anticoagulant drugs. In addition, the authors highlight that NTD dose adjustments may be useful in cases of gastrointestinal disorders [4]. The aim of the second study was to combine methotrexate (MTX) with gold nanoparticles (GNPs) to overcome the limitations of MTX, with in vitro and in vivo evaluations, in patients with rheumatoid arthritis. This study showed a marked improvement in the anti-arthritic effects of MTX when it was conjugated with GNPs. Furthermore, histological investigation supported these observations [5]. The aim of the third study was to study the IFN-regulated sialic-acid-binding Ig-like lectin 1 (SIGLEC-1) protein as a biomarker of disease phenotype, therapeutic response, and differential diagnosis in systemic sclerosis (SSc). Researchers have evaluated the expression levels of SIGLEC-1 on monocytes from 203 patients with SSc, 119 subjects with other rheumatic diseases, and 13 healthy controls (HS).

Patients with SSc had higher expressions of SIGLEC-1 on monocytes than HS, but significantly lower levels than patients with systemic lupus erythematosus (SLE) and mixed connective tissue disease (MCTD). However, the authors failed to find an association between SIGLEC-1 expression and fibrotic or vascular manifestations of the disease. This observational study reported that SIGLEC-1 expression on monocytes may be useful in differentiating SSc from other connective tissue diseases (e.g., SLE and MCTD), but not as a biomarker for SSc disease manifestations or activity [6]. The fourth article is a systematic review of the literature that examined the use of bronchoalveolar lavage (BAL) in systemic sclerosis–interstitial lung disease (SSc-ILD), focusing on the advantages and disadvantages of its real-life application. The authors included eighteen submissions in their evaluation and they observed positive correlations between lung function and BAL cytology and between BAL cellularity and high-resolution computed tomography (HRCT) findings. However, the researchers suggested the need for future studies to explain the true diagnostic potential and prognostic role of BAL in SSc-ILD [7]. The first objective of the fifth study was to review the current evidence on the correlation between gastro-oesophageal reflux disease (GERD) and idiopathic pulmonary fibrosis (IPF). In addition, the second objective was to review current studies related to treatments for GERD-IPF. The authors identified several studies that confirmed the association between GERD and IPF, with increased acid exposure, risk of gastric aspiration, and bile acid levels in these patients. Few studies have focused on the treatment of GERD, demonstrating that antacid therapy is unable to change the course of IPF. In conclusion, this review confirmed the correlation between GERD and IPF. However, further large prospective studies are needed to clarify these findings, with a specific focus on prevention and treatment strategies [8].

In this article, the authors described the disease course of two pediatric patients with idiopathic pulmonary hemosiderosis (IPH) treated with rituximab (RTX) for more than 4 years. These are the first two cases described with long-term follow-up of pediatric patients with IPH treated with RTX. RTX was effectively tolerated and prevented the occurrence of bleeding. In addition, RTX demonstrated a steroid-sparing effect, resulting in improved growth, lung function, and computed tomography abnormalities [9]. The seventh article is a case report of a 46-year-old male SSc patient with stable and extensive interstitial lung disease (ILD) who developed histologically documented pulmonary vasculopathy typical of pulmonary arterial hypertension (PAH) and received specific treatment for PAH as a bridge to transplantation. Interestingly, the authors documented the course of PH disease via right heart catheterization (RHC), with and without specific vasodilator therapies, which are essential in PAH, but not indicated and/or harmful in PH-ILD [10]. In the eighth article, the authors evaluated myofibroblastic differentiation markers of rheumatoid arthritis-like synoviocytes (RA-FLS) using ex vivo observations and in vitro evaluations following stimulation with TGF-β and IL-6. Second, they investigated the possible differentiation ability of rheumatoid arthritis synoviocytes. Second, they investigated the possible inhibitory role of tofacitinib, a JAK inhibitor, in this process. The study showed that the stimulation of TGF-β and IL-6 may play a role in mediating myofibroblast differentiation from RA-FLS by promoting collagen I and α-SMA and decreasing E-cadherin. In addition, the results showed that tofacitinib reduced the increase in collagen I and α-SMA in RA-FLS. However, the authors suggested the need for future studies to fully explore this topic and confirm the study results [11].

This Special Issue highlighted and presented recent findings on rheumatic diseases and highlighted strategies that can be used to combat the worsening symptoms of these diseases. In light of these diverse contributions, the importance of multidisciplinary teams using the expertise of laboratory researchers, clinicians, radiologists, and pathologists is undisputable. However, there are still many fundamental questions that remain unanswered, promising a great future for this field. We strongly encourage a wide group of readers to draw inspiration from this Special Issue and develop new approaches to the diagnosis and treatment of rheumatic diseases. Finally, the Guest Editors would like to sincerely thank all the authors and reviewers for their valuable contributions. We would also like to thank MDPI for deciding to publish this book and Ms. Fendy Fan for her kind assistance and technical support.

## Data Availability

Data sharing not applicable.
